# Predicting Survival Rates in Brain Metastases Patients from Non‐Small Cell Lung Cancer Using Radiomic Signatures Associated with Tumor Immune Heterogeneity

**DOI:** 10.1002/advs.202412590

**Published:** 2025-01-22

**Authors:** Fuxing Deng, Gang Xiao, Guilong Tanzhu, Xianjing Chu, Jiaoyang Ning, Ruoyu Lu, Liu Chen, Zijian Zhang, Rongrong Zhou

**Affiliations:** ^1^ The department of oncology Xiangya Hospital Central South University Changsha 410008 China; ^2^ National Clinical Research Center for Geriatric Disorders Xiangya Hospital Central South University Changsha 410008 China; ^3^ Xiangya Lung Cancer Center Xiangya Hospital Central South University Changsha 410008 China

**Keywords:** artificial intelligence, brain metastasis from NSCLC, immune heterogeneity, prognostic prediction, radiogenomics

## Abstract

Non‐small cell lung cancer (NSCLC) frequently metastasizes to the brain, significantly worsened prognoses. This study aimed to develop an interpretable model for predicting survival in NSCLC patients with brain metastases (BM) integrating radiomic features and RNA sequencing data. 292 samples are collected and analyzed utilizing T1/T2 MRIs. Bidirectional stepwise logistic regression is employed to identify significant variables, facilitating the construction of a prognostic model, which is benchmarked against four machine learning algorithms. BM tissue samples are processed for RNA extraction and sequencing. The optimal model achieved an AUC of 0.96 and a C‐index of 0.89 in the train set and an AUC of 0.84 with a C‐index of 0.78 in the test set, indicating strong predictive performance and generalizability. Patients from Xiangya Hospital are stratified into high‐risk (n = 11) and low‐risk (n = 30) groups. RNA sequencing revealed an enrichment of immune‐related pathways, particularly the interferon (IFN) pathway in the low‐risk group. Immune cell infiltration analysis identified a significant presence of CD8^+^‐T cells, IFNγ‐6/‐18 in the low‐risk group, suggesting an immunologically favorable tumor microenvironment. These findings highlight the potential of combining radiomic and RNA sequencing data for improved survival predictions and personalized treatment strategies in BM patients from NSCLC.

## Graphical abstract

Radiomic features extracted from T1/T2 MRI scans and integrative RNA sequencing data analysis yield an interpretable prognostic model predicting overall survival in non‐small cell lung cancer patients with brain metastases. This approach achieves robust predictive performance and identifies immune‐related pathways and immune infiltration, enabling personalized treatment strategies and improved clinical outcomes.

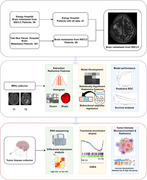

## 1 Introduction

Lung cancer is the leading cause of cancer‐related mortality globally, accounting for ≈1.6 million deaths annually.^[^ ^1^ ^]^ Non‐small cell lung carcinoma (NSCLC) constitutes the predominant form of lung cancer.^[^ ^2^ ^]^ In Shanghai, the reported crude and age‐adjusted incidences of NSCLC were 54.20 and 39.05 per 100000 people, respectively.^[^ ^3^ ^]^ The prognosis for NSCLC continues to be bleak, with a mere 26% of 5‐year overall survival (OS).^[^ ^4^, ^5^ ^]^ Brain metastases (BM) are a common complication in NSCLC patients and are associated with a generally poor prognosis. Despite advancements in diagnosing and treating metastatic brain tumors such as targeted therapies that cross the blood‐brain barrier and the use of stereotactic radiosurgery—the mortality and recurrence rates for BM remain high.^[^ ^6^, ^7^, ^8^ ^]^ Therefore, this highlights the urgent need for the development of innovative treatment strategies and prognostic models to improve outcomes for patients with NSCLC BM.

Recent breakthroughs in artificial intelligence (AI) have led to promising advances in prognostic research for various cancers.^[^ ^9^, ^10^ ^]^ Notably, AI and machine learning have been harnessed to discern the optimal prognostic indicators pertinent to brain tumors. For instance, a study involving BM utilized machine learning to predict the prognosis of BM patients with high accuracy.^[^ ^11^ ^]^ Another study constructed machine learning prognostic models to forecast the survival of BM from breast cancer patients. These models demonstrated high accuracy and outperformed traditional statistical methods in predicting survival.^[^ ^12^ ^]^ Radiomic and deep learning have shown promise in personalized medicine for immunotherapy in BM patients.^[^ ^13^ ^]^ Thus, these innovative technologies stand poised to enhance clinical decision‐making, facilitating precise diagnoses, identifying molecular markers, providing accurate prognostic predictions, and meticulously monitoring treatment responses.

Recent studies have highlighted the critical role of the brain tumor microenvironment (TME) in driving the progression of both primary and metastatic brain tumor.^[^ ^14^, ^15^ ^]^ The TME of BM differs significantly from that of extracranial lesions.^[^ ^16^ ^]^ Previous studies indicate that BM have a more immunosuppressive microenvironment than primary lung cancer.^[^ ^16^, ^17^ ^]^ Metastatic tumor cells are adept at manipulating immune responses and evading immune surveillance. They achieve this by secreting various tumor‐derived factors with immunosuppressive functions, including cytokines, chemokines, and extracellular vesicles.^[^ ^18^, ^19^, ^20^ ^]^ Understanding the immune characteristics of BM in NSCLC may also lead to the identification of new therapeutic targets and treatment mechanisms.

Currently, there is a lack of multicenter radiogenomics studies on prognostic prediction for BM patients from NSCLC. At the same time, the intricate relationship between radiogenomics prognostic models and immune characteristics warrants further investigation. The underlying immunological rationale for the opacity in the predictions of these models remains elusive. We conducted the first study to develop a radiogenomics model using magnetic resonance images (MRI) data from multiple hospitals and ribonucleic acid (RNA) sequencing, exploring the correlation between radiomic features and TME in BM patients from NSCLC.

## 2 Experimental Section

**Figure 1:**
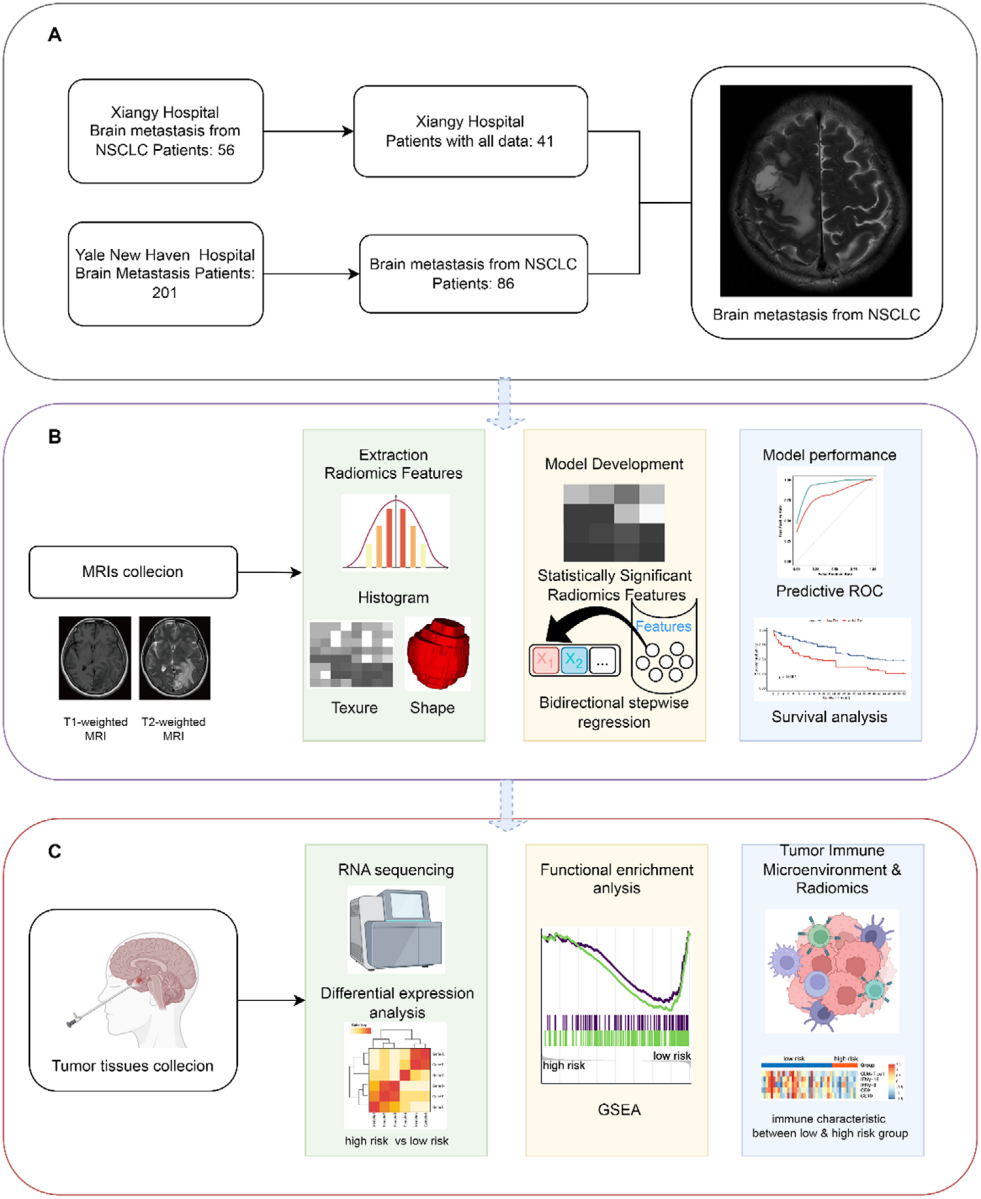
Overview of data collection, analysis workflow, and radiogenomics interpretation. This figure summarizes the study design, including patient screening, MRI‐based radiomic modeling, and radiogenomic integration for biological interpretation. A) Patient screening: Patients with BM from NSCLC were screened from Xiangya Hospital and Yale New Haven Hospital cohorts. Eligible patients with available brain MRI data were included for further analysis. B) MRI data acquisition and model development: Standardized MRI data underwent radiomic feature extraction and selection. Multiple algorithms were evaluated to construct a predictive model for OS. C) Radiogenomics and biological interpretation: Bulk RNA sequencing of BM was performed to investigate the biological relevance of radiomic features. Immunological differences between high‐ and low‐risk survival groups were analyzed, revealing distinct patterns of immune cell infiltration and their association with clinical outcomes.

The flow chart summarizes the study design (**Figure** **1**). The study employed a two‐cohort design for the development and validation of a prognostic model for BM patients from NSCLC. The first cohort (train set) included BM patients from Xiangya Hospital, while the second cohort (test set) consisted of patients from Yale New Haven Hospital. The approach incorporated MRI data, specifically T1‐ and T2‐weighted sequences, and RNA sequencing to develop a predictive model for OS. Integrative analyses of radiomics and RNA sequencing data identified risk groups with distinct TME, as visualized through comparative immune infiltration metrics. This approach aimed to identify radiogenomics markers in predicting clinical trajectories and risk stratification.

### 2.1 Images and Specimen Data

From 2021 to 2023, 56 cases of BM patients from NSCLC were sourced from Xiangya Hospital. Of these, 51 patients underwent BM surgery and sampling for sequencing, and 41 patients underwent preoperative MRI scanning for the train set. In order to ascertain whether the predictive model to be developed is capable of predicting the therapeutic effect of immunotherapy in patients, a further 22 BM patients from NSCLC who had undergone immunotherapy for targeting programmed death‐1/programmed death‐ligand‐1 were included in the verification process for the immunotherapy‐responded cohort. To ensure the validity of the radiomic analysis and the accuracy of the prognostic model, the following inclusion and exclusion criteria were applied to select a homogenous study population.

Inclusion Criteria: 1) Patients diagnosed with NSCLC exhibiting confirmed BM; 2) Patients had the RNA sequencing data from BM lesions; 3) Pre‐treatment brain MRI scans are available for radiomic analysis (e.g., T1/T2 sequences); 4) Sufficient clinical follow‐up data encompassing 36‐month OS.

Exclusion Criteria: 1) Subpar‐quality MRI scans (e.g., motion artifacts or incomplete images); 2) Patients lacking adequate clinical information, such as absent survival data or unverified diagnosis; 3) Instances where prior treatments (surgery and radiotherapy on brain lesions) significantly modified tumor morphology, thereby affecting radiomic analysis; 4) Presence of comorbidities or secondary malignancies significantly affecting overall survival, such as advanced cardiovascular diseases (e.g., recent myocardial infarction, congestive heart failure), severe trauma requiring hospitalization or surgery, chronic or uncontrolled systemic conditions (e.g., severe diabetes with complications, end‐stage renal disease).

Yale New Haven Hospital had 201 patients with BM for the test cohort, including 251 tissues from 86 cases of NSCLC patients with BM.^[^ ^21^ ^]^ The data included the T1 and the T2 magnetic resonance modality. Ethics approval was obtained from the Central South University Xiangya Hospital Research Ethics Committee (NO. 202210235).

MRI Equipment at Xiangya Hospital: All patients underwent a 3.0 T MRI scan (Siemens). The scanning parameters for the T1 sequences were as follows: repetition time (TR) = 472 ms and echo time (TE) = 12 ms. The parameters for the T2 sequences were TR = 4453 ms, and TE = 108 ms.

MRI Equipment at Yale New Haven Hospital: Scanner vendors included Siemens (158, 79%), General Electric (31, 15.5%), Philips (7, 3.5%), and Hitachi (4, 2%). The median echo time was 92.0 ms (10.0–400.0 ms), and the median repetition time was 9000.0 ms (1700.0–12000.0 ms).

### 2.2 Establishment of the Prognostic Survival Prediction Model

The model aimed to predict the survival of BM patients from NSCLC, utilizing radiomic features derived from MRI scans. The primary outcome focuses on predicting the survival of patients with BM from NSCLC for a minimum duration of 36 months. We employed the survival risk score generated by the predictive model as a radiomic signature to classify patients into two distinct groups: those at high risk of mortality and those at low risk of mortality.

#### 2.2.1 Extraction and Preprocessing of Radiomic Features

Regions of interests (ROIs) were manually delineated for 41 brain lesions for patients from Xiangya Hospital using the Insight Toolkit (ITK‐SNAP 3.8.0) across all slices for each sequence.^[^ ^22^ ^]^ The focus was exclusively on tumor tissue, excluding cystic, hemorrhagic, or necrotic areas. The boundaries of tumor tissue were determined by two sequences. In cases where a patient had multiple lesions larger than 3mm, all were delineated. These ROIs were independently defined and reviewed by two residents with over three years of training (F.D. and X.C.). Any discrepancies were validated by a third neuroradiologist with over 20 years of experience (R.Z.). All radiologists were blind to the survival status of BM patients.

#### 2.2.2 Model Development and Evaluation

In this study, we formulated a predictive model using a binary classification approach based on Logistic Regression. Initially, univariate logistic regression was performed as detailed in Table S1 (Supporting Information). Subsequently, the model was developed using the remaining statistically significant features (*P* < 0.05), through bidirectional stepwise regression, which utilized the Akaike Information Criterion (AIC) to balance model fit and complexity.^[^ ^23^ ^]^ The AIC is utilized to estimate predictive error and evaluate the relative quality of statistical models tailored to a specific dataset. To ensure the robustness of the selected features, a tenfold cross‐validation was conducted using the same bidirectional regression approach. The features were ranked according to how frequently they were chosen across the different folds, leading to the retention of the top four variables in the final model. For comparative assessment, we employed four additional techniques: Light Gradient Boosting Machine (Light GBM), a gradient boosting framework utilizing tree‐based learning algorithms;^[^ ^24^ ^]^ Support Vector Machine (SVM), a versatile supervised learning model effective in high‐dimensional spaces; Random Forest, an ensemble learning method that constructs multiple decision trees; and Multilayer Perceptron (MLP), a feedforward artificial neural network capable of distinguishing non‐linearly separable data. We also utilized the Cox regression model for survival analysis and comparison. To ensure the model's effectiveness and relevance for patients with multiple lesions, we used the masses that had been sequenced postoperatively to delineate and establish a prognostic model from Xiangya Hospital. By focusing on the most informative lesions, the team aims to mitigate inter‐tumor heterogeneity's confounding effects and create a more accurate and clinically applicable model. During the testing phase, every available tumor mass from each participant is meticulously analyzed. The assessment protocol involves a systematic evaluation of all tumor specimens per patient, with the ultimate goal of identifying the lesion that yields the highest predictive accuracy when used in the predictive OS model. The expected outcomes were evaluated using several statistical methods, including the Receiver Operating Characteristic (ROC) curve, the area under the ROC curve (AUC), the Concordance Index (C‐index), Decision Curve Analysis (DCA), and the Kaplan‐Meier (KM) curve. Additionally, metrics such as accuracy, sensitivity, and the F1‐score were also calculated to provide a comprehensive assessment. This comprehensive approach ensures robust and reliable prognostic modeling for clinical applications.

#### 2.2.3 Model Interpretability and Immune Differences

To address the interpretability of the model and further explore the immune differences between high‐ and low‐risk radiomic signature survival groups, we designed an integrated modeling framework to analyze radiomic and TME. Specifically, the strategy involves performing postoperative sequencing on precisely selected tumor nodules, which serve as the foundation for developing a radiomics and transcriptomics correlation model.

### 2.3 RNA Sequencing

BM tissues were collected and prepared into formalin‐fixed paraffin‐embedded (FFPE) samples. A total of 15 FFPE slides/samples were utilized, with a minimum tumor content of 20% confirmed by histological examination by pathologists. RNA was extracted from FFPE brain metastasis samples. The RIN value from samples was RIN value ≥ 6.5, 28S(25s)/18S ≥ 1.0. Libraries were prepared with mRNA poly‐A enrichment to isolate eukaryotic mRNA. RNA sequencing was performed using the DNBSEQ‐T7 platform. Sequencing was conducted in paired‐end mode. Each sample achieved a sequencing depth of over 100 million reads, with a mapping rate exceeding 90%.

#### 2.3.1 Differential Expression Analysis

Based on COUNT data of RNA sequencing, with a threshold of |log2FC| > 1 and adjusted *P* < 0.05, we performed differential expression analysis between radiomic signature groups (High risk group vs Low risk group) using the “DESeq2” R package.

#### 2.3.2 Functional Annotation

Gene set enrichment analysis (GSEA) was employed to investigate functional enrichment differences between radiomic signature groups, based on the Gene Ontology (GO) biological process, Kyoto Encyclopedia of Genes and Genomes (KEGG), and Hallmark gene sets. These gene sets were downloaded from the Molecular Signatures Database (MSigDB, v2023.1). Pathways meeting the threshold of |NES| > 1 and adjusted *P* < 0.05 were considered significantly enriched pathways.

Furthermore, we collected two gene sets related to tumor immune therapy response from Da‐Feng et al. study,^[^ ^25^ ^]^ including the interferon γ‐6 (IFNγ‐6) signature^[^ ^26^, ^27^ ^]^ and IFNγ‐18 signature.^[^ ^26^ ^]^ Based on these gene sets, we evaluated the score of each sample using the Gene Set Variation Analysis (GSVA) method.

#### 2.3.3 Tumor Immune Microenvironment Analysis

The Cell‐type Identification By Estimating Relative Subsets Of RNA Transcripts (CIBERSORT) and the Microenvironment Cell Populations‐counter algorithms were used to evaluate the infiltration of immune cells.^[^ ^28^, ^29^ ^]^ We collected previously published gene sets from the Charoentong et al. study, which contains 28 immune cells; then, the single sample gene set enrichment analysis algorithm was used to calculate the infiltration of 28 immune cells.^[^ ^30^ ^]^

EcoTyper is a machine learning framework designed to systematically identify cell states and cellular communities (ecotypes) using transcriptomic sequencing data.^[^ ^31^ ^]^ EcoTyper was used to perform the abundances of cell states and carcinoma ecotypes (CEs) through the EcoTyper framework (https://ecotyper.stanford.edu/) in BM from NSCLC. We explored the associations between the radiomic signature and cellular community structures.

### 2.4 Statistics Analysis

All MRI scans (T1, T2) underwent initial gradient nonlinearity correction and intensity normalization. This was followed by the application of the N4 bias field correction method to remove low‐frequency intensity non‐uniformities. After bias field correction, the images were resampled to a voxel size of 0.375 × 0.375 × 0.375. Feature extraction was carried out using PyRadiomics.^[^ ^32^ ^]^ In each sequence, 1144 radiomic features were extracted from each ROI. Data that follow a normal distribution are typically presented as the mean along with the standard deviation (SD). In contrast, data that do not conform to a normal distribution are represented by the median and the interquartile range. A significance threshold for the *P* value is conventionally set at 0.05 in statistical analyses. Statistical analysis was conducted using the statistical programming languages R (v4.1.0) and Python (v3.6). Feature extraction is implemented with the Python pyradiomics package (v3.0.1). The preprocessing of the MRI was performed using the Simple ITK package. All algorithms are available in the sklearn (v0.24.2) and lightgbm (v3.3.2) library.

## 3 Results

The study included a total of 127 patients with BM from NSCLC, 41 patients with 41 lesions from Xiangya Hospital, and 86 patients with 251 lesions from Yale New Haven Hospital. Baseline characteristics are presented in **Table** **1** and Table S2 (Supporting Information). The Yale New Haven cohort includes 86 patients, categorized by race as follows: 5 individuals identify as Asian or Pacific Islander, 5 as Black or African American, 69 as White, and 7 individuals have an unspecified race. The median age was 58.0 years (range, 31.0‐76.0) for Xiangya patients and 66.0 years (range, 34.0‐85.0) for Yale New Haven patients, respectively. Regarding gender distribution, females represented 41.5% of the Xiangya cohort and 65.1% of the Yale New Haven cohort. The majority of patients were non‐smokers (27 patients, 65.9%), while 14 patients (34.1%) had a history of smoking in the Xiangya cohort. A total of 25 patients (61.0%) were epidermal growth factor receptor or anaplastic lymphoma kinase (EGFR/ALK)‐positive, while 16 patients (39.0%) were EGFR/ALK‐negative. 19 patients (46.3%) had a Karnofsky Performance Status (KPS) score greater than 70, 16 patients (39.0%) had a score between 50 and 70, and 6 patients (14.6%) had a score below 50. Table S3 (Supporting Information) provides detailed information and the history of the treatment of each patient from Xiangya Hospital. Additionally, we included an immunotherapy cohort of 22 patients, with clinical data outlined in Table S4 (Supporting Information). In the Yale New Haven cohort, race did not show a statistically significant impact on OS, with a *P* value of 0.323 for comparisons between White and Black patients, and 0.991 for comparisons between White and Asian patients.

**Table 1:** Baseline of clinical data from Xiangya Hospital.

	Xiangya cohort(N = 41)
**Sex**	
Female	17 (41.5%)
Male	24 (58.5%)
**Age**	
Mean Standard Deviation	56.3 (11.6)
Median [Min, Max]	58.0 [31.0, 76.0]
**Smoking history**	
Non‐smoker	27 (65.9%)
Smoker	14 (34.1%)
**EGFR/ALK status**	
EGFR/ALK‐negative	16 (39.0%)
EGFR/ALK‐positive	25 (61.0%)
**Number of lesions**	
Solitary lesion	25 (61.0%)
Multiple lesions	16 (39.0%)
**KPS**	
80‐100	19 (46.3%)
50‐70	16 (39.0%)
<50	6 (14.6%)

### 3.1 Prediction Models

**Figure 2:**
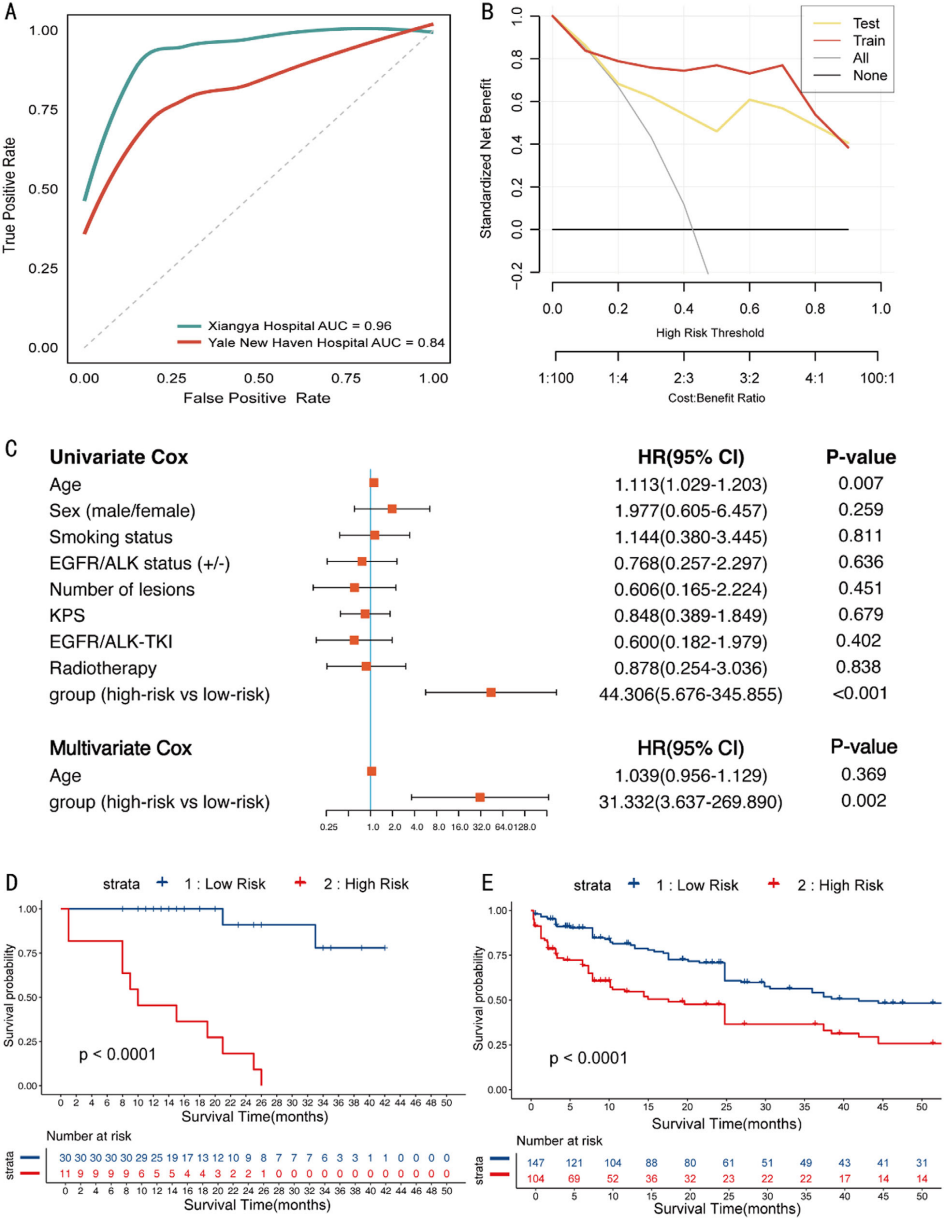
The results of prognostic prediction model among BM patients from NSCLC. A) ROCs of 36 months survival prediction, B) DCAs of the model of train set and test set; C) the forest plot of Univariate Cox and Multivariate Cox from Xiangya Hospital; D) KM curves of BM patients from Xiangya Hospital; E), KM curves of BM patients from Yale New Haven Hospital.

To identify robust predictors of prognosis in BM patients from NSCLC, we embarked on an extensive radiomic analysis using high‐dimensional radiomic features extracted from MRI scans. Univariate logistic regression analysis initially identified 70 statistically significant radiomic features (Table S5, Supporting Information). The four most prevalent variables were identified after tenfold cross‐validation and bidirectional stepwise regression (Table S6, Supporting Information). These features were further refined through a rigorous backward elimination process, resulting in the identification of four key radiomic biomarkers that effectively predicted patient survival outcomes: Log sigma 20 mm 3D Gray Level Run Length Matrix (GLRLM) Short Run Low Gray Level Emphasis from T1 MRI, Wavelet HHL(First H: High‐frequency components along the horizontal axis. Second H: High‐frequency components along the vertical axis. Third L: Low‐frequency components along the diagonal axis.) first order 90 Percentile from T1 MRI, Wavelet HHL first order Mean Absolute Deviation from T1 MRI, Wavelet HHL Gray Level Size Zone Matrix (GLSZM) Zone Entropy from T1 MRI. The details of four radiomics features are in **Table** **2**. These four selected features were subsequently utilized to construct a logistic regression‐based prognostic model. The performance of this model was evaluated using ROC curves and C‐index. In the train set, the model achieved an impressive AUC of 0.96 and a C‐index of 0.89, indicating excellent discrimination between patients with different survival (**Figure** **2**A). When tested on an independent validation cohort, the model maintained a commendable AUC of 0.84 and a C‐index of 0.78, indicating solid generalizability. The predictive OS model for BM patients from NSCLC demonstrated substantial accuracy in the training set from Xiangya Hospital, with a precision of 0.93 and a recall of 1.00, resulting in an F1 score of 0.97. In the Yale New Haven Hospital test set, the model exhibited comparable performance with precision, recall, and F1 scores of 0.79, 0.92, and 0.85, respectively (Table S7, Supporting Information).

**Table 2:** Detailed Radiomic Feature Descriptions.

Feature Name	Feature Type	Definition
Log Sigma 20 mm 3D GLRLM SRLGLE	Texture (GLRLM)	Quantifies short homogeneous runs of low‐intensity voxels after a 20 mm Gaussian filter.
Wavelet HHL First Order 90 Percentile	Intensity (First Order)	Represents the 90th percentile intensity value in the tumor post HHL wavelet transformation.
Wavelet HHL First Order MAD	Intensity (First Order)	Measures the mean absolute deviation of voxel intensities within the tumor post HHL transformation.
Wavelet HHL GLSZM Zone Entropy	Texture (GLSZM)	Quantifies the randomness in the size distribution of uniform intensity zones after HHL transformation.

Additional machine learning algorithms were employed, incorporating all 202 statistically significant radiomics features. However, these models did not outperform the logistic regression‐based model. The results for the test set were as follows: Light GBM: AUC = 0.52, MLP: AUC = 0.52, Random Forest: AUC = 0.51, and SVM: AUC = 0.50 (Figure S1, Supporting Information). Furthermore, a Cox proportional hazards model was developed using the same four radiomics features identified through logistic regression. Despite the rigorous feature selection process, the performance of the Cox model in the test set was suboptimal, with an AUC of 0.63 and a C‐index of 0.70. This indicates that while the radiomics features have predictive value, their integration into a survival analysis model requires further refinement to improve performance. The DCAs showed that the model outperformed non‐predictive thresholds, assuming all patients' survival situations starting at a threshold probability of 15% (Figure 2B).

To investigate whether the model is an independent prognostic factor in BM patients from NSCLC, we conducted univariate and multivariate Cox analyses of the model with clinical factors. The univariate Cox analysis revealed that a significant association between a higher radiomic signature and an adverse prognosis (*P* < 0.001; HR = 44.306, 95% CI: 5.676‐345.855, Figure 2C). Next, two factors (age and radiomic signature) with statistical significance in univariate Cox analysis were entered into multi‐variate Cox analysis (Figure 2C), which confirmed that the radiomic signature was an independent prognostic biomarker in BM patients from NSCLC in the Xiangya cohort (P = 0.002; HR = 31.332, 95% CI: 3.637‐269.890, Figure 2C). Similarly, univariate COX analysis in the testing cohort revealed that only the radiomic signature was statistically significant (*P* < 0.001; HR = 5.857, 95% CI: 2.934‐11.693, Figure S2, Supporting Information).

The Kaplan‐Meier (KM) survival curves illustrate the survival probability over time for BM patients from NSCLC, categorized into high‐risk and low‐risk patients based on the predictive model. Figure 2D presents the KM curves for survival data from BM patients from NSCLC at Xiangya Hospital. The KM curves demonstrates a distinct survival disparity between the two cohorts, with the low‐risk group consistently exhibiting higher median survival than the high‐risk group (*P* < 0.0001). Figure 2E illustrates the external test of the predictive model utilizing data from Yale New Haven Hospital. Similar to Figure 2D, the low‐risk group displays superior survival compared with the high‐risk group (*P* < 0.0001).

### 3.2 The Radiomic Signature Revealed Tumor Immune Heterogeneity

**Figure 3:**
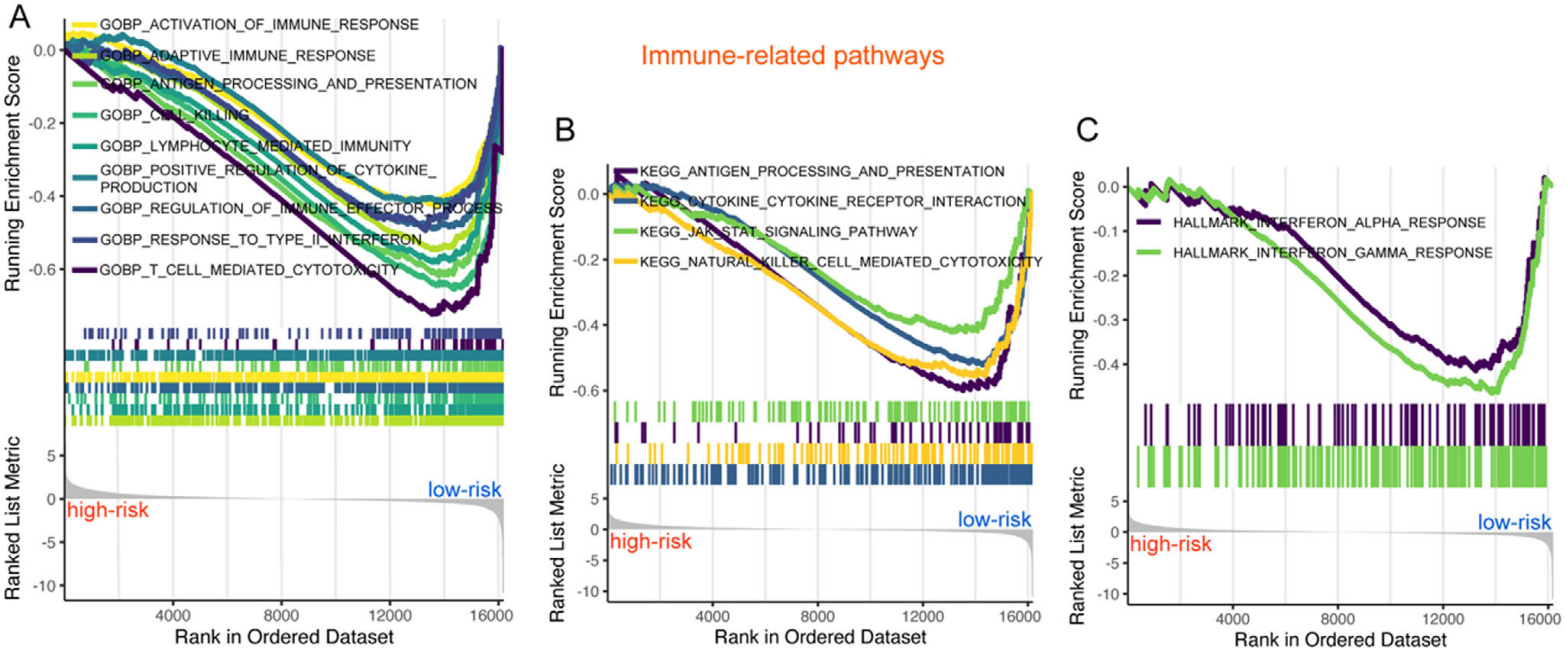
Immune‐related pathways are enriched in the low‐risk group. A) GO Biological Process, B) KEGG, and C) Hallmark enrichment analyses indicate that immune‐related pathways are enriched in the low‐risk group.

To explore the biological functions of this radiomic signature in BM from NSCLC, we conducted differential expression analysis using RNA sequencing data from 41 BM samples derived from NSCLC (including 30 low‐risk samples and 11 high‐risk samples). Using a threshold of |log2FC| > 1 and adjusted *P* < 0.05, we identified 31 upregulated genes and 23 downregulated genes (Figure S3A; Table S8, Supporting Information). Subsequently, we performed a functional enrichment analysis based on the GSEA method (Figure S3B–D, Supporting Information). We found various immune‐related pathways enriched in the low‐risk group, including Activation of the immune response, Adaptive immune response, Antigen processing and presentation, Cell killing, Lymphocyte mediated immunity, Positive regulation of cytokine production, Regulation of immune effector process, Response to type II interferon, T cell mediated cytotoxicity, Cytokine‐cytokine receptor interaction, JAK‐STAT signaling pathway, Natural killer cell mediated cytotoxicity, Interferon alpha response, and Interferon gamma response (**Figure** **3**A–C). We further examined the correlation between these immune‐related pathways and clinical outcomes. It was observed that pathways such as Cytokine‐cytokine receptor interaction, Natural killer cell mediated cytotoxicity, T cell mediated cytotoxicity, Lymphocyte mediated immunity, Regulation of immune effector process, Activation of the immune response, Positive regulation of cytokine production, Interferon gamma response, and Interferon alpha response were all associated with longer OS in BM patients from NSCLC (Figure S4, Supporting Information).

**Figure 4:**
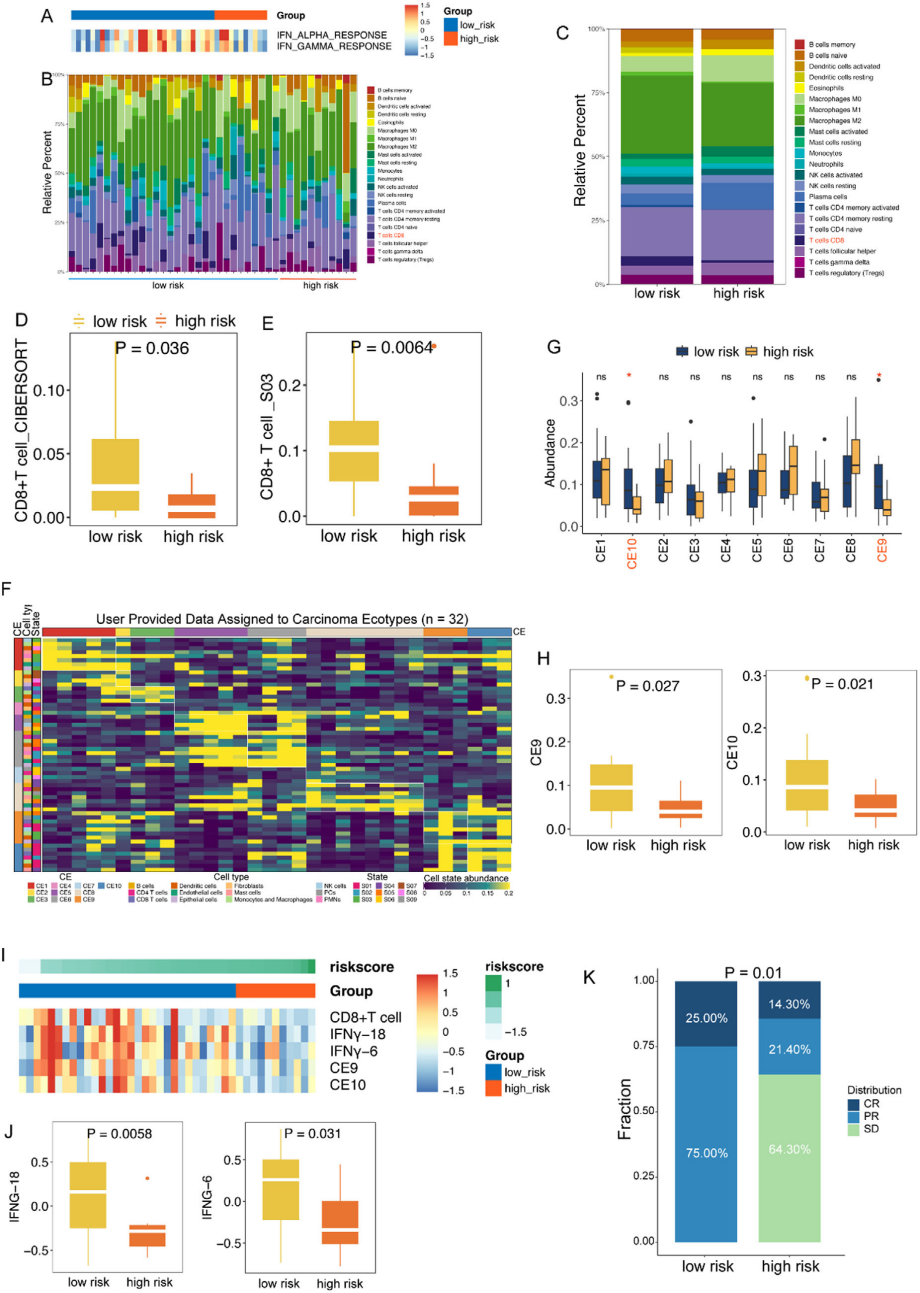
Immune characteristics of high‐ and low‐risk groups. A) The heatmap illustrates the differences in enrichment values of the IFN pathway between high‐ and low‐risk groups B,C) The fraction of the multiple cell types indicated proportional diversity between high‐ and low‐risk groups via the CIBERSORT algorithm. D) Compare the differences in the proportion of CD8+ T cells between the high‐ and low‐risk groups. E) Compare the differences in the state03 of CD8+ T cells between the high‐ and low‐risk groups. F) The distribution of cell state abundance across 10 carcinoma ecosystems (CEs). Only 32 samples assigned to CEs were shown, including 7 high‐risk and 25 low‐risk samples. G) Compare the differences in the composition of all CEs between high‐ and low‐risk groups. H) Compare the differences in the composition of CE9 and CE10 between high‐ and low‐risk groups. I) The heatmap illustrates the differences in enrichment values of the IFNγ‐6 and IFNγ‐18 between high‐ and low‐risk groups. J) Compare the differences in the score of the IFNγ‐6 and IFNγ‐18 between the high‐ and low‐risk groups. K) Compare the difference in the proportion of CR, PR, and SD between the high‐ and low‐risk groups.

Given the overall upregulation of immune‐related pathways in the low‐risk group, particularly the IFN‐related pathway (**Figure** **4**A), which plays a crucial role in antitumor immune responses,^[^ ^33^ ^]^ we then focused on the characteristics of the tumor immune microenvironment. We observed differences in immune cell infiltration among samples with different radiomic signature values (Figure 4B), particularly high infiltration of CD8+ T cells in the low‐risk group (*P* < 0.05; Figure 4C,D). This finding was validated through various immune cell infiltration algorithms (Figure S5, Supporting Information). We further investigated the differences in cell states and multicellular communities between the high‐risk and low‐risk groups. Regarding cell states, we found that the low‐risk group had higher infiltration of S03 state CD8+ T cells (*P* < 0.05; Figure 4E). Based on cell types and cell states, we constructed a multicellular community map and identified 10 carcinoma ecosystems (CEs). We observed a higher abundance of CE9 and CE10 in the low‐risk group (*P* < 0.05; Figure 4F–H). A previous study reported that CE9‐ and CE10‐high tumors exhibited proinflammatory characteristics and were closely linked to improved OS.^[^ ^31^ ^]^ Subsequently, we explored the correlation between immunotherapy response‐related markers and this radiomic signature. We found that the radiomic signature was significantly negatively correlated with tumor‐infiltrating CD8+ T cells, IFNγ signaling, and CE9/CE10 abundance (Figure 4I). The low‐risk group exhibited significantly higher expression of IFNγ‐6, IFNγ‐18, and CD8+ T cells (*P* < 0.05; Figure 4J). Moreover, we included an immunotherapy cohort comprising 22 NSCLC BM patients who received immunotherapy. After stratifying patients into high‐risk and low‐risk groups based on our radiomic signature, the difference of distribution in clinical response—complete response (CR), progressive response (PR), and stable disease (SD)—between low‐ and high‐risk group was found to be statistically significant (Figure 4K, *P* = 0.01). The low‐risk group had a higher proportion of CR (25% vs 14.3%) and PR (75% vs 21.4%) and a lower proportion of SD (0% vs 64.3%) compared to the high‐risk group (*P* = 0.01). Therefore, the low‐risk group may be associated with a better immunotherapy response in NSCLC BM. These results indicate that the radiomic signature may also capture features relevant to predicting treatment responses, providing a potential avenue for future studies.

## 4 Discussion

The objective of this study was to construct a predictive model for the survival of BM patients from NSCLC over a period exceeding three years, utilizing radiomic features extracted from MRI scans. We meticulously analyzed 292 lesions T1/T2 MRI from a cohort of 41 patients at Xiangya Hospital and 86 patients at Yale New Haven Hospital. By extracting radiomic features from these MRI images, we aimed to develop a prognostic prediction model of superior efficacy while simultaneously correlating the radiomic signature with the tumor immune microenvironment. A key strength of our dataset is the inclusion of multi‐center hospital patients, especially using brain tumor specimens for RNA‐sequencing, extensive clinical and molecular annotation, and long‐term follow‐up in most patients.

The predictive radiomic signature derived from our model was intricately linked to the tumor immune heterogeneity, reflecting biological traits and the immune infiltration of CD8+ T cells. This study represents a pivotal advancement in understanding and managing NSCLC with BM, addressing a significant gap in the existing literature on prognostic modeling for this patient group.^[^ ^12^ ^]^ BM from NSCLC are associated with poor survival rates, and their management remains a clinical challenge.^[^ ^34^ ^]^ Our model was able to discriminate patients with high‐risk and low‐risk survival among BM patients from NSCLC with AUCs of 0.96 in train set and 0.84 in the external test set. The systematic feature selection process enhances model interpretability, making it clinically accessible for oncologists. By identifying BM patients from NSCLC at high risk for adverse outcomes, our model can guide therapeutic decisions, such as considering immunotherapy, which may improve survival rates.

Currently, the development of a prognostic model for BM patients from NSCLC is a key research focus. Recent studies have explored biomarkers for risk stratification, including positron emission tomography/computerized tomography metabolic parameters and protein‐level markers like the Jacalin‐probed T‐antigen.^[^ ^35^, ^36^ ^]^ The Surveillance, Epidemiology, and End Results database has been used in AI modeling to create a predictive nomogram.^[^ ^37^ ^]^ Xu et al. combined a nomogram with MRI radiomic features to develop a prognostic model for BM patients from NSCLC receiving immunotherapy, achieving an AUC of 0.77.^[^ ^13^ ^]^ In patients with BM undergoing radiotherapy, there were statistically significant differences in age and KPS scores across different prognostic groups, and a combined prediction score was established. However, its predictive performance was not reported.^[^ ^38^ ^]^ Jeroen A. Crouzen and colleagues established graded prognostic assessment scores based on the public Lung‐molGPA 2022 data and Haaglanden Medical Center.^[^ ^39^ ^]^ For the prognostic assessment of BM patients from NSCLC, the one‐year prediction performance C‐index was 0.77, and the C‐index after two years was 0.71. Additionally, brain MRI has been employed to develop survival prediction models for gliomas and medulloblastomas, demonstrating the versatility of MRI in cancer prognosis.^[^ ^10^, ^40^ ^]^

Historically, few prognostic models for BM patients have offered sufficient interpretability. Previous studies have reported that MRI‐derived radiomics can serve as prognostic biomarkers for BM,^[^ ^13^, ^39^ ^]^ the biological characteristics of radiomics in BM remain unknown. In BM, a complex TME is formed through the interactions between cancer cells, immune cells, and resident cell types such as astrocytes.^[^ ^41^ ^]^ Our team previously identified that BM exhibit a unique TME compared to the primary lung cancer.^[^ ^17^ ^]^ Therefore, we explored the biological functions of radiomic signatures in BM and found that the overall upregulation of IFN response, increased infiltration of CD8+ T cells, and high abundance of CE9 and CE10 are observed in the low‐risk group. IFN, as a type of cytokine, serves as a critical mediator in the cellular communication processes within the TME, playing a crucial role in regulating cell states and activities.^[^ ^42^ ^]^ IFNγ exhibits both pro‐tumor and anti‐tumor activities. Typically, immune cells secrete IFNγ to exert anti‐tumor functions; CD8+ cytotoxic T lymphocytes are key producers of IFNγ and critical cells in the anti‐tumor immune process.^[^ ^33^, ^43^ ^]^ IFNγ may play a crucial role in immune therapy response. Tumors with high abundance of CE9 and CE10 were found to be pro‐inflammatory, exhibiting higher immune response activity and associated with longer OS. Furthermore, the multicellular ecosystem CE9, characterized by effector T cell components and IFNγ signaling, outperformed other cancer ecosystems in predicting better OS and responsiveness to immunotherapy.^[^ ^31^ ^]^

Immunotherapy has revolutionized the treatment of advanced NSCLC, demonstrating effective activity in metastatic cases and improving the 5‐year survival rate.^[^ ^44^, ^45^ ^]^ Our previous meta‐analysis found that NSCLC patients with BM who received immunotherapy had better outcomes than those who did not.^[^ ^46^ ^]^ Previous studies have also indicated a correlation between T cell signature/IFNγ and the response to cancer immunotherapy.^[^ ^25^, ^26^, ^27^, ^47^, ^48^ ^]^ Therefore, we explored the relationship between radiomic features and gene sets associated with predicting response to immunotherapy. We found that the radiomic signature was negatively correlated with these markers of immune activation. Meanwhile, we collected a cohort of 22 patients who had received immunotherapy to validate the ability of this radiomic signature to predict responses to immunotherapy. We found that the low‐risk group was more sensitive to immunotherapy, demonstrating the potential of radiomic features as predictors of response to immunotherapy. However, large prospective clinical studies are still needed for validation.

These findings suggest that MRI‐derived radiomic features could improve prognostic stratification, potentially based on immune heterogeneity in BM from NSCLC. We included the results of the radiomic feature selection process in Tables S2 and S3 (Supporting Information), which can serve as a resource for researchers identifying valuable features for prognostic survival modeling. The risk of overfitting is a well‐acknowledged challenge in high‐dimensional radiomic analyses. To mitigate this, we employed a multi‐step feature selection process, incorporating univariate logistic regression, bidirectional stepwise regression based on AIC, and tenfold cross‐validation. The final selection of four variables guarantees model simplicity, significantly diminishing the dimensionality of the data and reducing the likelihood of overfitting. Univariate and multivariate Cox regression analyses validated those clinical covariates, such as driver mutation status (e.g., EGFR mutation and ALK fusion), did not substantially influence the predictive value of the radiomic features.

The relationship between radiomic features and immune infiltration suggests that tumors with specific radiomic characteristics may harbor a more dynamic immune microenvironment. Non‐invasive radiomic biomarkers could facilitate personalized treatment strategies and improve prognostication. For example, texture features like Short Run Low Gray Level Emphasis and Zone Entropy may reflect tumor heterogeneity associated with immune cell infiltration or stromal components. The Wavelet HHL transformations capture high‐frequency spatial information, potentially indicating areas of necrosis, edema, or microvascular changes that accompany immune cell trafficking and activity. Understanding this interplay between radiomics biomarkers and immune dynamics bears significant clinical implications. Should certain radiomic features consistently signify an active immune microenvironment, they could prove instrumental in identifying patients poised to respond favorably to immunotherapies.^[^ ^49^ ^]^ This holds particular relevance for patients with BM, where procuring tissue samples for immune profiling presents considerable challenges. It is possible that tumors with more multiparametric imaging patterns possess a microenvironment conducive to immune cell infiltration, or that immune‐mediated tumor cell death contributes to the observed radiomics features.^[^ ^50^ ^]^ This is especially relevant for BM patients, where acquiring tissue samples for immune profiling is challenging. Further studies integrating advanced radiomic analytics with immunogenomic profiling are needed to clarify the relationships between immunotherapy and patient response.

The findings of this study hold significant implications for clinical practice, particularly in guiding personalized treatment plans for BM patients from NSCLC. By accurately predicting survival prognosis, our model enables the stratification of patients into high‐risk and low‐risk groups, each defined by distinct immune profiles. High‐risk patients, identified through the radiomic signature, could benefit from more aggressive treatments or closer monitoring, while low‐risk patients may receive less intensive interventions, optimizing resource allocation. Integrating radiomics into routine clinical workflows enhances the precision of prognostic assessments, facilitating more informed decision‐making and promoting individualized patient care. The radiomic signature scores, derived as non‐invasive biomarkers, present a compelling alternative to biopsy‐based immune profiling. This method allows for repeated evaluations throughout the treatment process, enabling dynamic patient stratification and real‐time modifications to therapeutic strategies as tumor‐immune interactions evolve. These advancements underscore the potential of radiomics as a valuable tool in the era of precision medicine, aligning with the growing emphasis on individualized patient care.^[^ ^51^ ^]^ Furthermore, integrating gene correlation results that link radiomic features to specific gene pathways enhances the model's interpretability, increasing confidence among clinicians and patients. For future clinical applications, it would be beneficial to include additional clinical covariates, integrate multi‐omics data for more robust modeling, and seek prospective validation to optimize the model's effectiveness in therapeutic decision‐making.^[^ ^52^ ^]^

Despite the promising results, this study has several limitations that warrant consideration. First, the sample size, although larger than many previous studies, is still relatively not large, particularly when considering the subset of patients who underwent both MRI scanning and sequencing. Additionally, the Yale New Haven dataset lacked detailed information on treatment regimens and driver gene mutations, which limited our ability to comprehensively investigate the interactions between radiomic features, treatment effects, and outcomes. Moving forward, obtaining thorough treatment data will be crucial for developing predictive treatment‐response models tailored to specific therapeutic modalities, such as immunotherapy, thereby enhancing both the accuracy and clinical relevance of these models. Lastly, the follow‐up period was limited to 36 months, which may not fully capture the long‐term outcomes. Future research should aim to include larger, more diverse cohorts and extend the follow‐up period to further validate and refine our predictive models.

## 5 Conclusion

This study highlights the potential of radiomics features extracted from MRI scans to predict the 36‐month survival of BM patients from NSCLC. By comparing logistic regression and other machine learning algorithms, we identified significant predictive variables, achieving promising predictive performance. Moreover, the integration of multi‐modal radiomic and RNA‐sequencing data underscores the potential of radiomics features to reflect variations in the immune landscape, which could be critical for tailoring immunotherapy strategies.

## Conflict of Interest

The authors declare no conflict of interest.

## Author Contributions

F.D. and G.X. contributed equally to this work. F.D. conceptualized the study; created the methodology and software; accessed, verified, validated, visualized, and analyzed the data; and wrote the manuscript. G.X. conceptualized the study, curated the data, investigated patient history, acquired resources, and wrote the manuscript. X.C., G.T., and J.N. accessed and curated the data, investigated patient history, and acquired resources. L.C., Z.Z., and R.L. validated the data. R.Z. supervised and acquired funding. All authors had full access to all the data in the study and had final responsibility for the decision to submit for publication.

## Supporting information



Supporting Information

Supplemental Table 1

## Data Availability

Data are available upon reasonable request to zhourr@csu.edu.cn. The raw sequence data reported in this paper have been deposited in the Genome Sequence Archive (Genomics, Proteomics & Bioinformatics 2021) ref. [53] in National Genomics Data Center (Nucleic Acids Res 2022) ref. [54], China National Center for Bioinformation/Beijing Institute of Genomics, Chinese Academy of Sciences (GSA‐Human: HRA003286) that are publicly accessible at https://ngdc.cncb.ac.cn/gsa‐human.

## References

[1] J. Ferlay , I. Soerjomataram , R. Dikshit , S. Eser , C. Mathers , M. Rebelo , D. M. Parkin , D. Forman , F. Bray , Int. J. Cancer 2015, 136, E359. 25220842 10.1002/ijc.29210

[2] F. Bray , M. Laversanne , H. Sung , J. Ferlay , R. L. Siegel , I. Soerjomataram , A. Jemal , CA Cancer J. Clin. 2024, 74, 229. 38572751 10.3322/caac.21834

[3] H. Fan , Z. Y. Shao , Y. Y. Xiao , Z. H. Xie , W. Chen , H. Xie , G. Y. Qin , N. Q. Zhao , BMJ Open 2015, 5, e009419. 10.1136/bmjopen-2015-009419 PMC4691760 26700282

[4] A. K. Ganti , A. B. Klein , I. Cotarla , B. Seal , E. Chou , JAMA Oncol. 2021, 7, 1824. 34673888 10.1001/jamaoncol.2021.4932 PMC8532041

[5] B. S. Matos , S. Peixoto da Silva , M. H. Vasconcelos , C. P. R. Xavier , Cancer Drug Resist. 2024, 7, 19. 38835347 10.20517/cdr.2024.04 PMC11149106

[6] A. Steindl , T. J. Brunner , K. Heimbach , K. Schweighart , G. M. Moser , H. M. Niziolek , E. Moor , J. Kreminger , A. M. Starzer , K. Dieckmann , B. Gatterbauer , G. Widhalm , M. Preusser , A. S. Berghoff , Eur. J. Cancer 2022, 162, 170. 34998049 10.1016/j.ejca.2021.12.005

[7] J. H. Suh , R. Kotecha , S. T. Chao , M. S. Ahluwalia , A. Sahgal , E. L. Chang , Nat. Rev. Clin. Oncol. 2020, 17, 279. 32080373 10.1038/s41571-019-0320-3

[8] W. J. Liu , L. Wang , F. M. Zhou , S. W. Liu , W. Wang , E. J. Zhao , Q. J. Yao , W. Li , Y. Q. Zhao , Z. Shi , J. G. Qiu , B. H. Jiang , Drug Resist. Update 2023, 70, 100987. 10.1016/j.drup.2023.100987 37392558

[9] A. Chelliah , D. A. Wood , L. S. Canas , H. Shuaib , S. Currie , K. Fatania , R. Frood , C. Rowland‐Hill , S. Thust , S. J. Wastling , S. Tenant , C. McBain , K. Foweraker , M. Williams , Q. Wang , A. Roman , C. Dragos , M. MacDonald , Y. H. Lau , C. A. Linares , A. Bassiouny , A. Luis , T. Young , J. Brock , E. Chandy , E. Beaumont , T. C. Lam , L. Welsh , J. Lewis , R. Mathew , et al., Neuro Oncol. 2024, 26, 1138. 38285679 10.1093/neuonc/noae017 PMC11145448

[10] R. Verma , T. J. Alban , P. Parthasarathy , M. Mokhtari , P. Toro Castano , M. L. Cohen , J. D. Lathia , M. Ahluwalia , P. Tiwari , Sci. Adv. 2024, 10, eadi0302. 39178259 10.1126/sciadv.adi0302 PMC11343024

[11] L. Liu , W. Che , B. Xu , Y. Liu , J. Lyu , Y. Zhang , Neurosurg. Rev. 2024, 47, 296. 38922516 10.1007/s10143-024-02519-5

[12] N. F. Marko , Z. Xu , T. Gao , M. W. Kattan , R. J. Weil , Cancer 2012, 118, 3749. 22180078 10.1002/cncr.26716

[13] J. Xu , P. Wang , Y. Li , X. Shi , T. Yin , J. Yu , F. Teng , Front. Immunol. 2024, 15, 1373330. 38686383 10.3389/fimmu.2024.1373330 PMC11057328

[14] D. F. Quail , J. A. Joyce , Cancer Cell 2017, 31, 326. 28292436 10.1016/j.ccell.2017.02.009 PMC5424263

[15] K. E. de Visser , J. A. Joyce , Cancer Cell 2023, 41, 374. 36917948 10.1016/j.ccell.2023.02.016

[16] Y. Kudo , C. Haymaker , J. Zhang , A. Reuben , D. Y. Duose , J. Fujimoto , S. Roy‐Chowdhuri , L. M. Solis Soto , H. Dejima , E. R. Parra , B. Mino , R. Abraham , N. Ikeda , A. Vaporcyan , D. Gibbons , J. Zhang , F. F. Lang , R. Luthra , J. J. Lee , C. Moran , J. T. Huse , H. Kadara , I. I. Wistuba , Ann. Oncol. 2019, 30, 1521. 31282941 10.1093/annonc/mdz207 PMC6771224

[17] G. Xiao , L. Li , G. Tanzhu , Z. Liu , X. Gao , X. Wan , D. Xiao , L. Chen , X. Xia , R. Zhou , J. Immunother Cancer 2023, 11, e006243. 36868569 10.1136/jitc-2022-006243 PMC9990629

[18] M. Ahmadi , R. Abbasi , J. Rezaie , Cell Commun. Signaling 2024, 22, 9. 10.1186/s12964-023-01370-3 PMC10763406 38167133

[19] Y. Liu , X. Cao , J. Mole. Med. 2016, 94, 509. 10.1007/s00109-015-1376-x 26689709

[20] X. Hu , X. Deng , J. Xie , H. Zhang , H. Zhang , B. Feng , Y. Zou , C. Wang , Pharmaceuticals (Basel) 2024, 17, 850. 39065701 10.3390/ph17070850 PMC11280367

[21] D. Ramakrishnan , L. Jekel , S. Chadha , A. Janas , H. Moy , N. Maleki , M. Sala , M. Kaur , G. C. Petersen , S. Merkaj , M. von Reppert , U. Baid , S. Bakas , C. Kirsch , M. Davis , K. Bousabarah , W. Holler , M. Lin , M. Westerhoff , S. Aneja , F. Memon , M. S. Aboian , Scientific Data 2024, 11, 254. 38424079 10.1038/s41597-024-03021-9 PMC10904366

[22] P. A. Yushkevich , Y. Gao , G. Gerig , in *38th Annual Int. Conf. IEEE Eng. Med. Biol. Soc*., IEEE, Orlando, FL, USA, 2016, pp. 3342–3345. 10.1109/EMBC.2016.7591443 PMC5493443 28269019

[23] M. Wang , J. Wright , A. Brownlee , R. Buswell , Energy Build. 2016, 127, 313.

[24] G. Ke , Q. Meng , T. Finley , T. Wang , W. Chen , W. Ma , Q. Ye , T.‐Y. Liu , Adv. Neural Information Proc. Syst. 2017, 30, https://proceedings.neurips.cc/paper/2017/hash/6449f44a102fde848669bdd9eb6b76fa‐Abstract.html.

[25] D. F. Lin , H. L. Li , T. Liu , X. F. Lv , C. M. Xie , X. M. Ou , J. Guan , Y. Zhang , W. B. Yan , M. L. He , M. Y. Mao , X. Zhao , L. Z. Zhong , W. H. Chen , Q. Y. Chen , H. Q. Mai , R. J. Peng , J. Tian , L. Q. Tang , D. Dong , J. Natl. Cancer Inst. 2024, 116, 1294. 38637942 10.1093/jnci/djae081

[26] M. Ayers , J. Lunceford , M. Nebozhyn , E. Murphy , A. Loboda , D. R. Kaufman , A. Albright , J. D. Cheng , S. P. Kang , V. Shankaran , S. A. Piha‐Paul , J. Yearley , T. Y. Seiwert , A. Ribas , T. K. McClanahan , J. Clin. Invest. 2017, 127, 2930. 28650338 10.1172/JCI91190 PMC5531419

[27] F. Newell , I. Pires da Silva , P. A. Johansson , A. M. Menzies , J. S. Wilmott , V. Addala , M. S. Carlino , H. Rizos , K. Nones , J. J. Edwards , V. Lakis , S. H. Kazakoff , P. Mukhopadhyay , P. M. Ferguson , C. Leonard , L. T. Koufariotis , S. Wood , C. U. Blank , J. F. Thompson , A. J. Spillane , R. P. M. Saw , K. F. Shannon , J. V. Pearson , G. J. Mann , N. K. Hayward , R. A. Scolyer , N. Waddell , G. V. Long , Cancer Cell 2022, 40, 88. 34951955 10.1016/j.ccell.2021.11.012

[28] B. Chen , M. S. Khodadoust , C. L. Liu , A. M. Newman , A. A. Alizadeh , Methods Mol. Biol. 2018, 1711, 243. 29344893 10.1007/978-1-4939-7493-1_12 PMC5895181

[29] E. Becht , N. A. Giraldo , L. Lacroix , B. Buttard , N. Elarouci , F. Petitprez , J. Selves , P. Laurent‐Puig , C. Sautès‐Fridman , W. H. Fridman , A. de Reyniès , Genome Biol. 2016, 17, 218. 27765066 10.1186/s13059-016-1070-5 PMC5073889

[30] P. Charoentong , F. Finotello , M. Angelova , C. Mayer , M. Efremova , D. Rieder , H. Hackl , Z. Trajanoski , Cell Rep. 2017, 18, 248. 28052254 10.1016/j.celrep.2016.12.019

[31] B. A. Luca , C. B. Steen , M. Matusiak , A. Azizi , S. Varma , C. Zhu , J. Przybyl , A. Espín‐Pérez , M. Diehn , A. A. Alizadeh , M. van de Rijn , A. J. Gentles , A. M. Newman , Cell 2021, 184, 5482. 34597583 10.1016/j.cell.2021.09.014 PMC8526411

[32] J. J. Van Griethuysen , A. Fedorov , C. Parmar , A. Hosny , N. Aucoin , V. Narayan , R. G. Beets‐Tan , J.‐C. Fillion‐Robin , S. Pieper , H. J. Aerts , Cancer Res. 2017, 77, e104. 29092951 10.1158/0008-5472.CAN-17-0339 PMC5672828

[33] A. M. Gocher , C. J. Workman , D. A. A. Vignali , Nat. Rev. Immunol. 2022, 22, 158. 34155388 10.1038/s41577-021-00566-3 PMC8688586

[34] M. A. Vogelbaum , P. D. Brown , H. Messersmith , P. K. Brastianos , S. Burri , D. Cahill , I. F. Dunn , L. E. Gaspar , N. T. N. Gatson , V. Gondi , J. T. Jordan , A. B. Lassman , J. Maues , N. Mohile , N. Redjal , G. Stevens , E. Sulman , M. van den Bent , H. J. Wallace , J. S. Weinberg , G. Zadeh , D. Schiff , J. Clin. Oncol. 2021, 40, 492. 34932393 10.1200/JCO.21.02314

[35] L. Guan , W. Wang , X. Ji , H. Cheng , W. Du , L. Ye , Ann. Clin. Trans. Neurol. 2024, 11, 1765. 10.1002/acn3.52082 PMC11251471 38721992

[36] Y. Feng , B. Cheng , S. Zhan , H. Liu , J. Li , P. Chen , Z. Wang , X. Huang , X. Fu , W. Ye , R. Wang , Q. Wang , Y. Xiang , H. Wang , F. Zhu , X. Zheng , W. Fu , G. Hu , Z. Chen , J. He , W. Liang , Eur. J. Nucl. Med. Mol. Imaging 2024, 51, 3400. 38722381 10.1007/s00259-024-06740-8 PMC11369054

[37] Z. Lin , R. Wang , Y. Zhou , Q. Wang , C. Y. Yang , B. C. Hao , C. F. Ke , Ann. Transl. Med. 2022, 10, 16. 35242861 10.21037/atm-21-6295 PMC8825534

[38] I. Chambrelant , L. Kuntz , C. Le Fèvre , D. Jarnet , J. Jacob , G. Noël , Cancers (Basel) 2024, 16, 2602. 39061240 10.3390/cancers16142602 PMC11275202

[39] J. A. Crouzen , M. E. Mast , M. Hakstege , M. L. D. Broekman , C. Baladi , B. J. A. Mertens , R. D. S. Nandoe Tewarie , M. Kerkhof , M. J. Vos , K. W. Maas , E. T. D. Souwer , R. G. J. Wiggenraad , N. van der Voort van Zyp , M. Kiderlen , A. L. Petoukhova , J. D. Zindler , Radiother. Oncol. 2024, 198, 110405. 38925263 10.1016/j.radonc.2024.110405

[40] Y. J. Wang , P. Wang , Z. Yan , Q. Zhou , F. Gunturkun , P. Li , Y. Hu , W. E. Wu , K. Zhao , M. Zhang , H. Lv , L. Fu , J. Jin , Q. Du , H. Wang , K. Chen , L. Qu , K. Lin , M. Iv , H. Wang , X. Sun , H. Vogel , S. Han , L. Tian , F. Wu , J. Gong , Cancer Cell 2024, 42, 1239. 38942025 10.1016/j.ccell.2024.06.002 PMC13010562

[41] M. R. Strickland , C. Alvarez‐Breckenridge , J. F. Gainor , P. K. Brastianos , Cancer Discov. 2022, 12, 1199. 35394521 10.1158/2159-8290.CD-21-0976 PMC11440428

[42] D. J. Propper , F. R. Balkwill , Nat. Rev. Clin. Oncol. 2022, 19, 237. 34997230 10.1038/s41571-021-00588-9

[43] S. J. Szabo , B. M. Sullivan , C. Stemmann , A. R. Satoskar , B. P. Sleckman , L. H. Glimcher , Science 2002, 295, 338. 11786644 10.1126/science.1065543

[44] I. Eguren‐Santamaria , M. F. Sanmamed , S. B. Goldberg , H. M. Kluger , M. A. Idoate , B. Y. Lu , J. Corral , K. A. Schalper , R. S. Herbst , I. Gil‐Bazo , Clin. Cancer Res. 2020, 26, 4186. 32354698 10.1158/1078-0432.CCR-20-0798

[45] J. R. Brahmer , J. S. Lee , T. E. Ciuleanu , R. Bernabe Caro , M. Nishio , L. Urban , C. Audigier‐Valette , L. Lupinacci , R. Sangha , A. Pluzanski , J. Burgers , M. Mahave , S. Ahmed , A. J. Schoenfeld , L. G. Paz‐Ares , M. Reck , H. Borghaei , K. J. O'Byrne , R. G. Gupta , J. Bushong , L. Li , S. I. Blum , L. J. Eccles , S. S. Ramalingam , J. Clin. Oncol. 2023, 41, 1200. 36223558 10.1200/JCO.22.01503 PMC9937094

[46] X. Chu , L. Niu , G. Xiao , H. Peng , F. Deng , Z. Liu , H. Wu , L. Yang , Z. Tan , Z. Li , R. Zhou , Front. Immunol. 2022, 13, 875488. 35693805 10.3389/fimmu.2022.875488 PMC9175180

[47] C. R. Bolen , R. McCord , S. Huet , G. M. Frampton , R. Bourgon , F. Jardin , P. Dartigues , E. A. Punnoose , E. Szafer‐Glusman , L. Xerri , P. Sujobert , G. Salles , J. M. Venstrom , Blood Adv. 2017, 1, 1884. 29296835 10.1182/bloodadvances.2016000786 PMC5728140

[48] C. S. Grasso , J. Tsoi , M. Onyshchenko , G. Abril‐Rodriguez , P. Ross‐Macdonald , M. Wind‐Rotolo , A. Champhekar , E. Medina , D. Y. Torrejon , D. S. Shin , P. Tran , Y. J. Kim , C. Puig‐Saus , K. Campbell , A. Vega‐Crespo , M. Quist , C. Martignier , J. J. Luke , J. D. Wolchok , D. B. Johnson , B. Chmielowski , F. S. Hodi , S. Bhatia , W. Sharfman , W. J. Urba , C. L. Slingluff Jr. , A. Diab , J. Haanen , S. M. Algarra , et al., Cancer Cell 2021, 39, 122. 33306984 10.1016/j.ccell.2020.11.015 PMC7885306

[49] A. B. Levine , L. Nobre , A. Das , S. Milos , V. Bianchi , M. Johnson , N. R. Fernandez , L. Stengs , S. Ryall , M. Ku , M. Rana , B. Laxer , J. Sheth , S. G. Sbergio , I. Fedorakova , V. Ramaswamy , J. Bennett , R. Siddaway , U. Tabori , C. Hawkins , Nat. Commun. 2024, 15, 5790. 38987542 10.1038/s41467-024-49595-1 PMC11237052

[50] E. Hoffmann , M. Masthoff , W. G. Kunz , M. Seidensticker , S. Bobe , M. Gerwing , W. E. Berdel , C. Schliemann , C. Faber , M. Wildgruber , Nat. Rev. Clin. Oncol. 2024, 21, 428. 38641651 10.1038/s41571-024-00891-1

[51] R. J. Gillies , P. E. Kinahan , H. Hricak , Radiology 2016, 278, 563. 26579733 10.1148/radiol.2015151169 PMC4734157

[52] J. Xie , X. Deng , Y. Xie , H. Zhu , P. Liu , W. Deng , L. Ning , Y. Tang , Y. Sun , H. Tang , M. Cai , X. Xie , Y. Zou , MedComm 2020, 5, e502. 10.1002/mco2.502 PMC10901283 38420162

[53] T. Chen , X. Chen , S. Zhang , et al., Genom Proteom. Bioinf. 2021, 19, 578.

[54] CNCB‐­ NGDC Members and Partners , Nucleic Acids Res. 2022, 50, D27.34718731

