# Supplementary material for: Predicting Survival Rates in Brain Metastases Patients from Non‐Small Cell Lung Cancer Using Radiomic Signatures Associated with Tumor Immune Heterogeneity
Source: Adv Sci (Weinh). 2025 Jan 22;12(10):2412590. doi: 10.1002/advs.202412590 (PMC11904944; doi:10.1002/advs.202412590)
Supplement: Supplementary file 1 — Supporting Information [file ADVS-12-2412590-s001.docx]

Supplementary Materials for

Predicting Survival Rates In Brain Metastases Patients From Non-Small Cell Lung Cancer Using Radiomic Signatures Associated With Tumor Immune Heterogeneity

Fuxing Deng^1,⸸^, Gang Xiao^1,⸸^, Guilong Tanzhu^1^，Xianjing Chu^1^, Jiaoyang Ning^1^,Ruoyu Lu^1^,Liu Chen^1^, Zijian Zhang^1^, Rongrong Zhou^1,2,3*^

^1^ The department of oncology, Xiangya Hospital, Central South University; Changsha, China

^2^ National Clinical Research Center for Geriatric Disorders, Xiangya Hospital, Central South University

^3^ Xiangya Lung Cancer Center, Xiangya Hospital, Central South University

⸸ Fuxing Deng and Gang Xiao contributed equally to this work.

**Correspondence to:** Rongrong Zhou, the department of oncology of Xiangya Hospital, Central South University, 410008, Changsha, China. [**zhourr@csu.edu.cn**](mailto:zhourr@csu.edu.cn)**;**

**This Word file includes**

**Supplementary Figure S1 to Figure S5:**


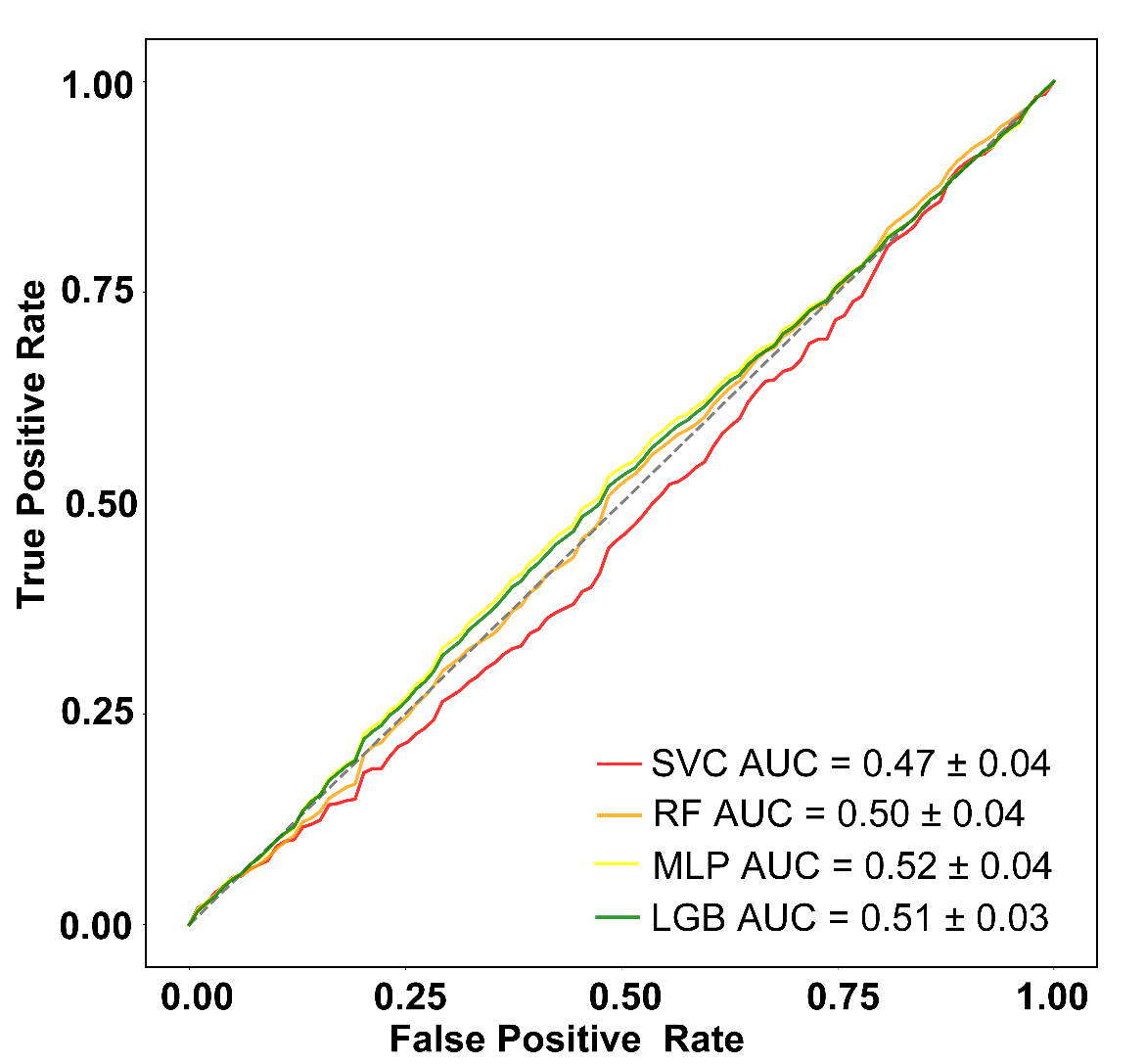
**Figure S1.** Performance of four machine learning algorithms on the test Set.


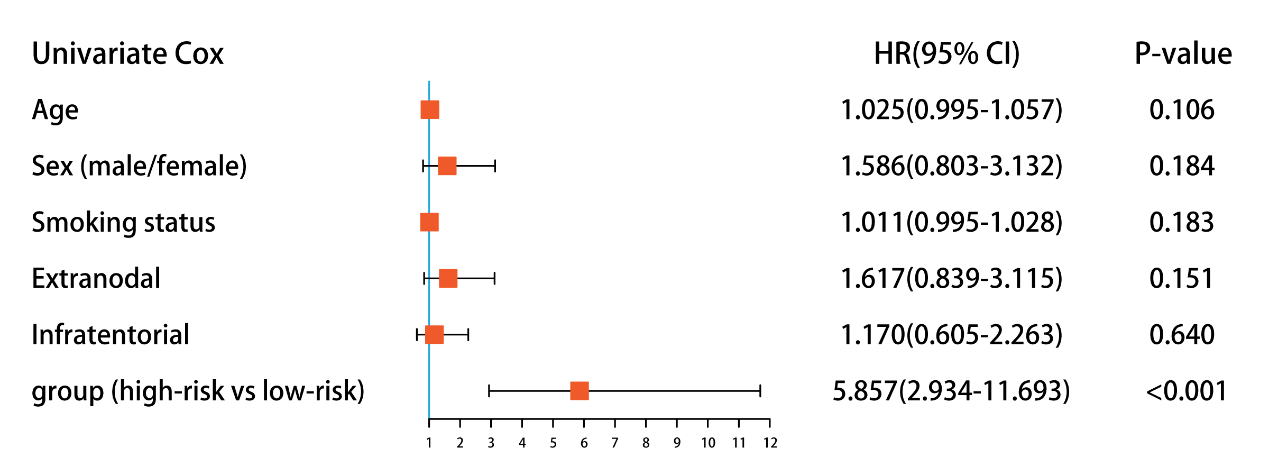
**Figure S2.** The forest plot of Univariate Cox from Yale New Haven Hospital.


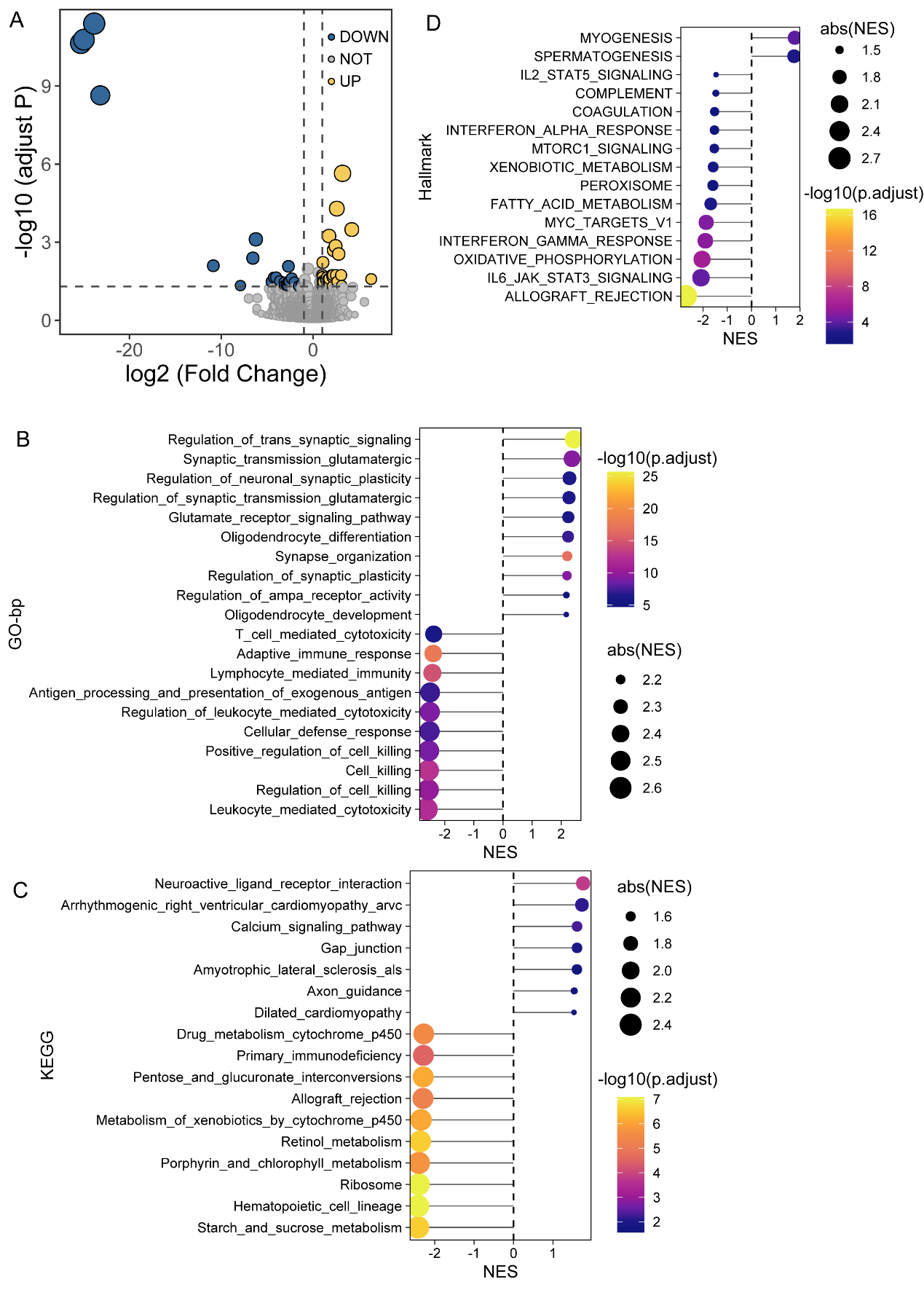
**Figure S3.** The functional difference between low- and high-risk groups. A) Volcano plot showing differentially expressed genes between low- and high-risk groups. Gene set enrichment analysis (GSEA) for analyzing GOBP(B), KEGG (C), and Hallmark (D) between low- and high-risk groups.


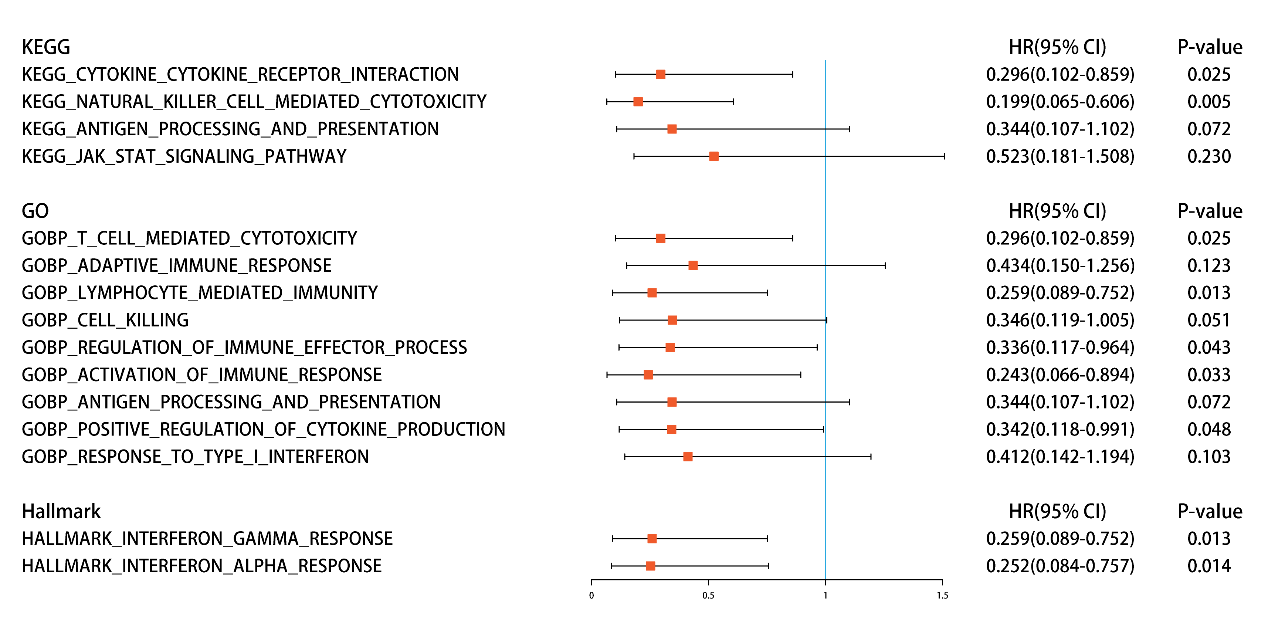
**Figure S4.** The correlation between immune-related pathways and clinical outcomes. Forest plots showed that immune-related pathways enriched by GOBP, KEGG, and Hallmark were associated with better clinical outcomes.


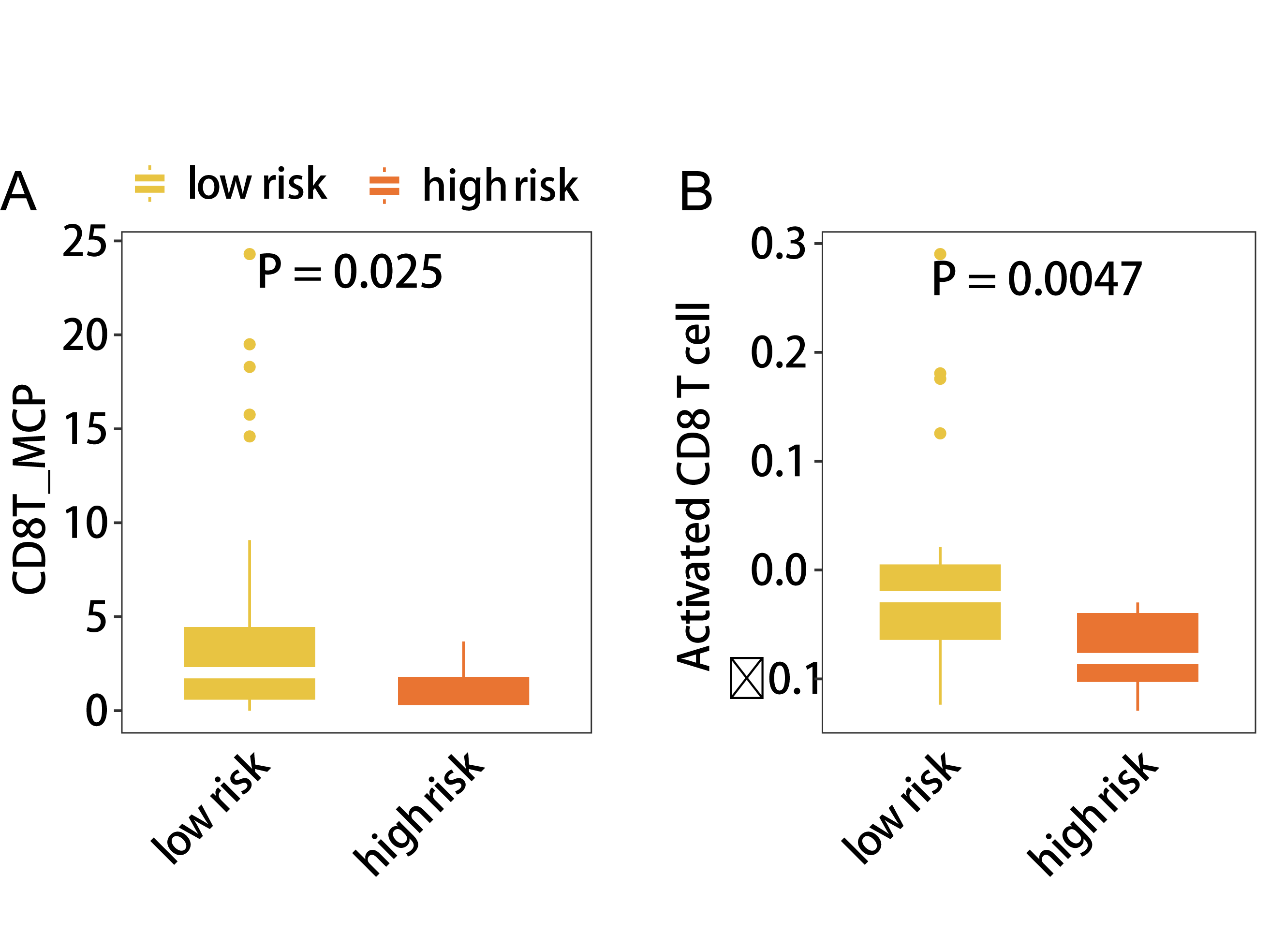
**Figure S5.** The infiltration of CD8+T cells. Comparison of CD8+ T cell infiltration differences between high-risk and low-risk groups by MCPCOUNTER (A) and ssgsea (B) methods.
